# Anstrengungsinduzierte Anaphylaxie beim Vogel‐Ei‐Syndrom: Die Rolle von Eigelb‐ und Vogelallergenen

**DOI:** 10.1111/ddg.15852_g

**Published:** 2025-10-23

**Authors:** Andrea Nolting, Peter Schmid‐Grendelmeier, Carole Guillet

**Affiliations:** ^1^ Allergiestation Dermatologische Klinik Universitätsspital Zürich Zürich Schweiz; ^2^ Medizinische Fakultät Universität Zürich Zürich Schweiz; ^3^ Christine Kühne – Center for Allergy Research and Education (CK‐CARE) Davos Schweiz

Sehr geehrte Herausgeber,

Eine 18‐jährige Patientin wurde uns zur Abklärung nach einer vermuteten anstrengungsinduzierten anaphylaktischen Reaktion zugewiesen. Etwa eine Stunde nach dem Verzehr einer Quiche mit Schafskäse ging sie joggen. Nach etwa 10 Minuten entwickelte sie zunächst trockenen Husten und Schwitzen, gefolgt von Atemnot und generalisierter Urtikaria. Es wurde kein Notfall aufgesucht, da sich die Atemnot zeitnah besserte und die übrigen Symptome innerhalb von 8 Stunden abklangen. Die Patientin empfand das Ereignis aber insgesamt als sehr bedrohlich.

Bereits seit einigen Jahren klagte sie über gastrointestinale Beschwerden (Krämpfe, Durchfall) und orales Jucken nach Mahlzeiten, konnte jedoch keine konkreten Auslöser identifizieren. Im Anschluss an das oben beschriebene Ereignis begann sie ein Ernährungstagebuch zu führen. Dabei vermutete sie Ei als Auslöser, da sie Symptome nach dem Verzehr sowohl von rohem als auch gekochtem Ei – einschließlich Eiweiß und Eigelb – feststellte.

Die Patientin hatte eine bekannte Katzenhaarallergie, verneinte jedoch Heuschnupfensymptome. Sie wuchs mit Wellensittichen und Kaninchen auf und berichtete über Niesen und juckende Augen beim Kontakt mit dem Vogelkäfig. Weitere Vorerkrankungen waren nicht bekannt. Es erfolgte eine allergologische Testung mittels Pricktest auf verschiedene Pollen, Hausstaubmilben, Schimmelpilze sowie auf Eiweiß, Eigelb und Vogelfedern. Positive Reaktionen zeigten sich auf Eiweiß, Eigelb, Gesamt‐Ei‐Extrakt, Hausstaubmilben und einige Pollen. Die Ergebnisse der Pricktests auf Eiweiß, Eigelb und Gesamt‐Ei‐Extrakt sind in Abbildung 1 dargestellt. Serologisch fand sich ein erhöhter Gesamt‐IgE‐Wert (172 kU/l) sowie spezifisches IgE gegen Eiweiß und Eigelb (α‐Livetin, Gal d5). Das Sensibilisierungsmuster ist in Tabelle 1 dargestellt.

**ABBILDUNG 1:**
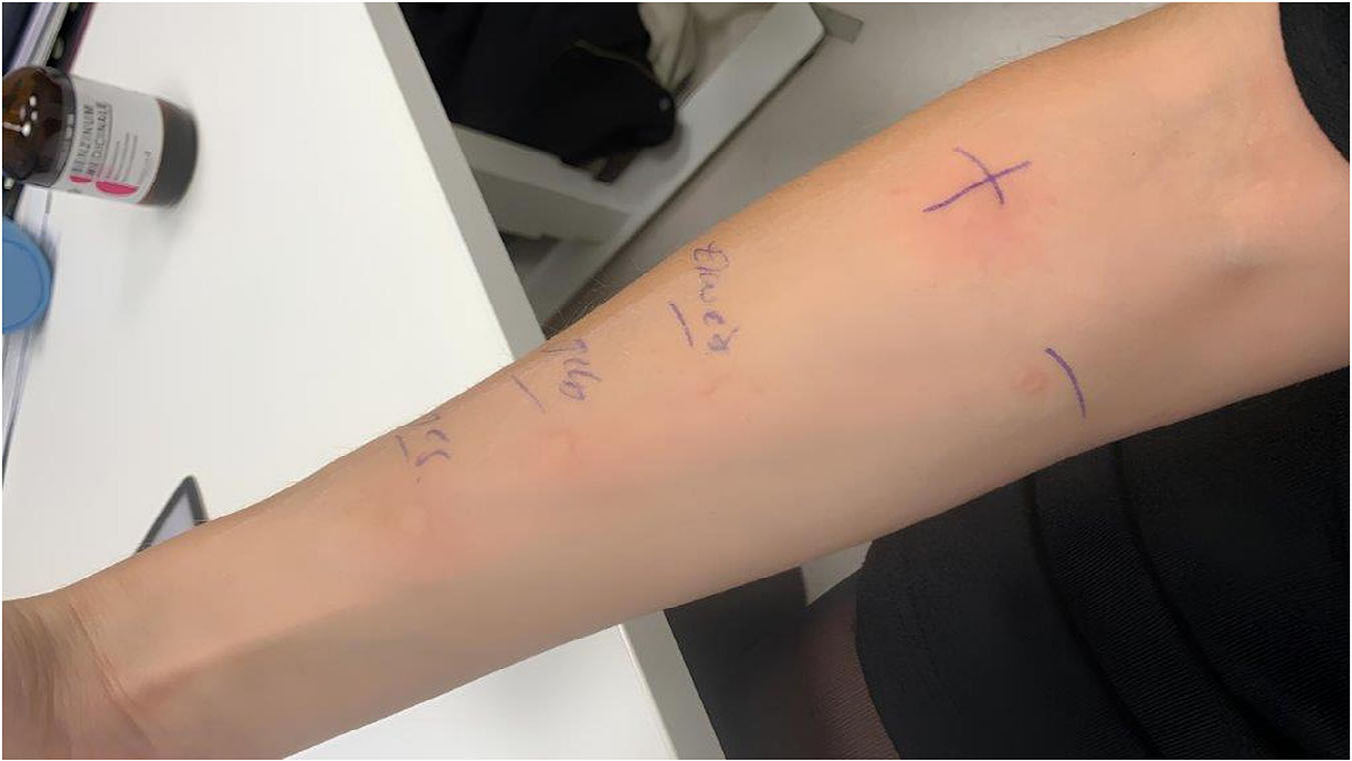
Pricktestung mit verschiedenen Bestandteilen des Hühnereis. Positive Reaktionen zeigten sich in der Standardtestung von oben nach unten auf Eiklar, Eigelb und Gesamt‐Ei‐Extrakt sowie auf Histamin als Positivkontrolle (mit einem „+“ markiert).

**TABELLE 1:** Serologische Abklärung bei einer 18‐jährigen Patientin mit Hühnereiallergie.

Parameter	Result
Gesamt‐IgE	172 kU/l
Gesamt‐Ei‐Extrakt	3.9 kU/l (NL:< 0.1 kU/l)
Ovomucoid (Gal d1)	<0.1 kU/l
Ovalbumin (Gal d2)	<0.1 kU/l
Eigelb (Gal d5)	0.56 kU/l
Hausstaubmilben	
*Dermatophagoides pteronyssinus*	8.29 kU/l
*Dermatophagoides farinae*	18.5 kU/l
Pollen	
Gräserpollen	1.45 kU/l
Birkenpollen	2.56 kU/l

Spezifisches IgE gegen Ovomucoid (Gal d1) und Ovalbumin (Gal d2) war jedoch nicht erhöht. Eine Testung auf Allergien gegen Geflügel, Milch oder Weizen wurde nicht durchgeführt, da die Patientin diese Nahrungsmittel problemlos vertrug.

Aufgrund der typischen Symptome nach Eikonsum, positiver Hauttestung sowie Nachweis spezifischer IgE‐Antikörper wurde eine Hühnereiallergie mit möglicher anstrengungsinduzierter Anaphylaxie diagnostiziert. Der Nachweis von IgE gegen Gal d5 und das Auftreten von rhinokonjunktivalen Symptomen bei Kontakt mit Wellensittichen weisen auf ein mögliches Vogel‐Ei‐Syndrom hin – trotz negativer Hauttestung auf Vogelfedern und beschwerdefreiem Konsum von Geflügelfleisch. Der Patientin wurde geraten, alle Nahrungsmittel, die Eiweiß oder Eigelb enthalten, strikt zu meiden. Sie wurde über Anaphylaxie‐Symptome, mögliche Auslöser und Sofortmaßnahmen aufgeklärt – einschließlich des Verzichts auf rohe und gekochte Eier sowie Spuren von Ei in verarbeiteten Lebensmitteln. Ein Notfallset mit Antihistaminika und Kortikosteroiden wurde verordnet. Zusätzlich wurde zur Mitführung eines Adrenalin‐Autoinjektors geraten. Eine orale Immuntherapie wurde nicht empfohlen, da diese derzeit überwiegend Kindern vorbehalten ist.^1^, ^2^

Eine Hühnereiallergie bei Erwachsenen ist selten, mit einer Prävalenz von etwa 0,1 %. Bei Kindern hingegen ist sie die zweithäufigste Nahrungsmittelallergie und bildet sich häufig bis zum Jugendalter zurück.^3^, ^4^ Die Symptome sind variabel, betreffen jedoch häufig den Gastrointestinaltrakt, können aber auch Hautreaktionen oder schwere anaphylaktische Reaktionen einschließen.^5^ Das Eiweiß enthält vier Hauptallergene: Ovomucoid (Gal d1), Ovalbumin (Gal d2), Ovotransferrin (Gal d3) und Lysozym (Gal d4). Im Eigelb sind vor allem das Hühnerei‐Serumalbumin (Gal d5) und YGP42 (Gal d6) die Hauptallergene.^6^

Bei unserer Patientin fanden sich erhöhte IgE‐Werte für das Gesamt‐Ei‐Extrakt sowie spezifisches IgE gegen Gal d5, was auf eine Eigelb‐Allergie hinweist. Obwohl die typischen Eiweißallergene nicht erhöht waren, spricht die klinische Symptomatik auch für eine Eiweißallergie, was auf eine mögliche Sensibilisierung gegenüber weiteren Allergenen hinweist.

Neuere Studien zeigen, dass eine Eigelb‐Allergie mit erhöhtem IgE gegen Gal d5 mit einer Sensibilisierung gegen Geflügelfleisch und Vogelfedern – dem sogenannten Vogel‐Ei‐Syndrom – assoziiert sein kann.^2^ Unsere Patientin vertrug Geflügelfleisch problemlos, berichtete jedoch über rhinokonjunktivale Beschwerden beim Kontakt mit dem Vogelkäfig. Dies könnte als möglicher Sensibilisierungsweg für die Eigelb‐Allergie interpretiert werden.

Anaphylaxien können durch verschiedene Faktoren verstärkt werden, zum Beispiel durch körperliche Anstrengung, Stress, Infektionen, Medikamente oder Alkohol.^7^ Obwohl ein Belastungstest zur Diagnosesicherung bei anstrengungsabhängiger Nahrungsmittelanaphylaxie als Goldstandard gilt, wurde in diesem Fall aufgrund der klaren zeitlichen Zuordnung der Symptome zum Eikonsum sowie der möglichen Belastung und des Risikos für die Patientin darauf verzichtet. Zudem traten bei der Patientin auch ohne körperliche Anstrengung milde Symptome auf, was die primäre Diagnose einer Hühnereiallergie weiter stützt.

Die Diagnose einer Hühnereiallergie erfordert erhebliche diätetische Umstellungen, die zu eingeschränkter Nahrungsmittelauswahl, reduzierter Lebensqualität und erhöhter Angst vor allergischen Reaktionen führen können.^8^ Angesichts der zunehmenden Prävalenz von Nahrungsmittelallergien im Erwachsenenalter – einschließlich Ei‐Allergien – spielen Aufklärung, klare Kennzeichnung und Prävention eine zentrale Rolle, um anaphylaktische Zwischenfälle zu vermeiden, die Lebensqualität der Betroffenen zu verbessern und Gesundheitskosten zu senken.

## DANKSAGUNG

Open access publishing facilitated by Universitat Zurich, as part of the Wiley ‐ Universitat Zurich agreement via the Consortium Of Swiss Academic Libraries.

## INTERESSENKONFLIKT

P.S.‐G. erhielt Honorare für Vorträge und Beratungstätigkeiten von Euroimmun und ThermoFisher Diagnostics. A.N. und C.G. erklären, dass keine potenziellen Interessenkonflikte bestehen.
